# A31 OUTCOMES OF PATIENTS UNDERGOING REPEAT VIDEOCAPSULE ENDOSCOPY (VCE) PRESENTING WITH OBSCURE GASTROINTESTINAL BLEEDING (OGIB)

**DOI:** 10.1093/jcag/gwab049.030

**Published:** 2022-02-21

**Authors:** T Krahn, M I Suliman, B Halloran, S Wasilenko, S Zepeda-Gomez

**Affiliations:** Gastroenterology, University of Alberta, Edmonton, AB, Canada

## Abstract

**Background:**

Videocapsule Endoscopy (VCE) as well as balloon-assisted enteroscopy (BAE) are useful tools in the diagnosis and management of obscure gastrointestinal bleeding (OGIB). There is limited data assessing the diagnostic yield of VCE in subjects with OGIB according to different subtypes: 1) obscure overt 2) inactive overt and 3) active overt.

**Aims:**

We evaluated the diagnostic yield and outcomes of patients undergoing a second VCE in OGIB.

**Methods:**

This is a retrospective analysis of all patients who underwent more than one VCE completed at the University of Alberta from January 1, 2015 to August 31, 2021. Demographic and background information was collected including previous endoscopy results, cross-sectional imaging, and subsequent interventions. Patient data was analyzed according to subtype of OGIB at presentation.

**Results:**

During the study period, there were 59 subjects who met inclusion criteria. The indication for VCE was recurrent iron deficiency anemia (IDA), active OGIB, and/or inactive OGIB in 38 subjects. The median time to VCE after initial endoscopic evaluation was 61 days. Median age of cases was 61.5 years and 49% were female.

Initial VCE had clinically significant positive findings in 68% of cases (Table 1). The diagnostic yield was 75%, 56% and 74% in active OGIB, inactive OGIB, and IDA, respectively. Active bleeding or fresh blood was present in 33% of VCEs for active OGIB compared to 11% of inactive OGIB and 13% of subjects with IDA.

At second VCE investigation, there were positive findings in 42% of cases. Findings on second VCE differed from initial VCE in 17 of 38 cases. Findings that changed clinical management were found in 76% of patients after first and second VCE.

BAEs were performed in 20 subjects after VCE, with therapeutics applied in 75%. The most common findings were arteriovenous malformations (AVMs) (65%) and erosions/ulcers (15%).

**Conclusions:**

The diagnostic yield of VCE is high in appropriately selected patients and did not significantly differ in patients with IDA, active, and inactive OGIB. Serial VCE is appropriate for the investigation of suspected recurrent small bowel bleeding when initial workup is nondiagnostic.

**Funding Agencies:**

None

**Table:** Table 1: Comparison of findings after 1st and 2nd VCE

		Active OGIB (n=12)	Inactive OGIB (n=9)	IDA (n=23)
1st VCE	Complete	100%	78%	91%
	Source identified	75	56	74
	AVM	25	33	48
	Erosions/ulcers	42	11	17
	Mass		11	4
	Fresh blood	33	11	13
	BAE	50	56	48
BAE with treatment	25	33	26
2nd VCE	Complete	83	89	100
	Negative findings	42	78	43
	AVM	25	22	43
	Erosions/ulcers	8		9
	BAE	8	33	35
BAE with treatment		22	9

